# Supplementary material for: Active Vertex Model for cell-resolution description of epithelial tissue mechanics
Source: PLoS Comput Biol. 2017 Jun 30;13(6):e1005569. doi: 10.1371/journal.pcbi.1005569 (PMC5493290; doi:10.1371/journal.pcbi.1005569)
Supplement: S1 Appendix — (PDF) [file pcbi.1005569.s001.pdf]

Active Vertex Model for Cell-Resolution Description of Epithelial Tissue Mechanics

Daniel L. Barton, Silke Henkes, Cornelis J. Weijer and Rastko Sknepnek

S1 APPENDIX: FORCE ON THE CELL CENTRE

In this appendix we derive the expression for forces on the cell centre, Eq. (7) in the main text, starting from the expression for the Vertex Model (VM) energy given in Eq. (1) in the main text. The force on cell i is computed as the negative derivative with respect to $\mathbf{r}_i$ of the energy functional E_{VM} (Eq. (1) in the main text). Conceptually, this is a straightforward calculation with the only real complication being that E_{VM} is most naturally written in terms of the positions of the Voronoi vertices, $\mathbf{r}_\mu$, while in the AVM we track positions of cell centres. In general,

$$\mathbf{F}_i = -\nabla_{\mathbf{r}_i} E_{VM}. \tag{1}$$

When computing the gradient in the last expression we need to keep in mind that moving particle i changes the shapes of all of its neighbouring cells. Therefore, moving cell i exerts a force on a number of cell centres in its surrounding. All those contributions have to be taken into account when computing the force $\mathbf{F}_i$. A direct consequence of this coupling between the cell and all of its neighbouring cells is that the force $\mathbf{F}_i$ *cannot* be written as a simple sum of pairwise interactions between cell i and each of its neighbours. We'll get back to this point below.

We start by computing the direct contributions resulting from moving particle i itself. The area term will produce a force

$$\mathbf{F}_i^{area} = -\nabla_{\mathbf{r}_i} \frac{K_i}{2}\left(A_i - A_i^0\right)^2 = -K_i\left(A_i - A_i^0\right)\nabla_{\mathbf{r}_i} A_i. \tag{2}$$

We need to compute $\nabla_{\mathbf{r}_i} A_i \equiv \frac{\partial A_i}{\partial \mathbf{r}_i}$, which we write by using the vector form of the chain rule for the calculating derivatives,

$$\left[\nabla_{\mathbf{r}_i} A_i\right]_k = \sum_{\nu\in\Omega_i} \left[\nabla_{\mathbf{r}_\nu} A_i\right]_m \left[\frac{\partial \mathbf{r}_\nu}{\partial \mathbf{r}_i}\right]_{mk}, \tag{3}$$

where the sum is over all vertices surrounding particle i, referred to as the *loop* Ω_i of particle i. $\left[\frac{\partial \mathbf{r}_\nu}{\partial \mathbf{r}_i}\right]$ is the 3×3 Jacobian matrix of the transformation between coordinates of cell centres and positions of the vertices of the dual Voronoi tessellation. $[\ldots]_k$ represents the $k^{\text{th}}-$component of the gradient vector, with $k\in\{x,y,z\}$ and we assume summation over the repeated index m. The Jacobian can be computed using the barycentric coordinates that connect centres and vertices introduced in Eq. (5) and Fig. 2 (both in the main text). We have

$$\frac{d\mathbf{r}_\mu}{d\mathbf{r}_p} = \mathbf{r}_i \otimes \frac{d}{d\mathbf{r}_p}\left(\frac{\lambda_1}{\Lambda}\right) + \frac{\lambda_1}{\Lambda}\delta_{ip}\hat{I} + \mathbf{r}_j \otimes \frac{d}{d\mathbf{r}_p}\left(\frac{\lambda_2}{\Lambda}\right) + \frac{\lambda_2}{\Lambda}\delta_{jp}\hat{I} + \mathbf{r}_k \otimes \frac{d}{d\mathbf{r}_p}\left(\frac{\lambda_3}{\Lambda}\right) + \frac{\lambda_3}{\Lambda}\delta_{kp}\hat{I}. \tag{4}$$

where

$$\begin{aligned}\lambda_1 &= l_i^2\left(l_j^2 + l_k^2 - l_i^2\right)\\ \lambda_2 &= l_j^2\left(l_k^2 + l_i^2 - l_j^2\right)\\ \lambda_3 &= l_k^2\left(l_i^2 + l_j^2 - l_k^2\right),\end{aligned} \tag{5}$$

are the barycentric coordinates and $\Lambda = \lambda_1 + \lambda_2 + \lambda_3$ with

$$\begin{aligned}l_i &= \left|\mathbf{r}_j - \mathbf{r}_k\right| = \left|\mathbf{r}_{jk}\right|\\ l_j &= \left|\mathbf{r}_k - \mathbf{r}_i\right| = \left|\mathbf{r}_{ki}\right|\\ l_k &= \left|\mathbf{r}_i - \mathbf{r}_j\right| = \left|\mathbf{r}_{ij}\right|.\end{aligned} \tag{6}$$

$\hat{I}$ is a 3×3 identity matrix, and $\otimes$ stands for the outer product between two vectors. We proceed by calculating

$$\frac{d}{d\mathbf{r}_p}\left(\frac{\lambda_q}{\Lambda}\right) = \frac{1}{\Lambda^2}\left(\Lambda \frac{d\lambda_q}{d\mathbf{r}_p} - \lambda_q \frac{d\Lambda}{d\mathbf{r}_p}\right), \tag{7}$$

for $q = 1, 2, 3$. It is straightforward to show that

$$\frac{d\lambda_q}{d\mathbf{r}_p} = \left(L^2 - 4l_q^2\right)\frac{d\left(l_q^2\right)}{d\mathbf{r}_p} + l_q^2 \frac{d\left(L^2\right)}{d\mathbf{r}_p}, \tag{8}$$

where l_q is defined in Eq. (6) and $L^2 = l_1^2 + l_2^2 + l_3^2$. We readily calculate,

$$\frac{d\left(l_i^2\right)}{d\mathbf{r}_p} = \begin{cases} 0 & \text{for } p = i \\ 2\mathbf{r}_{jk} & \text{for } p = j \\ -2\mathbf{r}_{jk} & \text{for } p = k \end{cases}, \tag{9}$$

$$\frac{d\left(l_j^2\right)}{d\mathbf{r}_p} = \begin{cases} -2\mathbf{r}_{ki} & \text{for } p = i \\ 0 & \text{for } p = j \\ 2\mathbf{r}_{ki} & \text{for } p = k \end{cases}, \tag{10}$$

$$\frac{d\left(l_k^2\right)}{d\mathbf{r}_p} = \begin{cases} 2\mathbf{r}_{ij} & \text{for } p = i \\ -2\mathbf{r}_{ij} & \text{for } p = j \\ 0 & \text{for } p = k \end{cases}, \tag{11}$$

where $\mathbf{r}_{ij} = \mathbf{r}_i - \mathbf{r}_j$, etc. Combining the last three expressions gives

$$\frac{d\left(L^2\right)}{d\mathbf{r}_p} = \begin{cases} 2\left(-\mathbf{r}_{ki} + \mathbf{r}_{ij}\right) & \text{for } p = i \\ 2\left(\mathbf{r}_{jk} - \mathbf{r}_{ij}\right) & \text{for } p = j \\ 2\left(-\mathbf{r}_{jk} + \mathbf{r}_{ki}\right) & \text{for } p = k \end{cases}. \tag{12}$$

In order to reduce the computational effort, it is convenient to precompute and store derivatives for all three values of $p = i, j, k$. We start with the case $p = i$ for which we have,

$$\frac{d\lambda_1}{d\mathbf{r}_i} = 2\left|\mathbf{r}_{jk}\right|^2\left(-\mathbf{r}_{ki} + \mathbf{r}_{ij}\right). \tag{13}$$

$$\frac{d\lambda_2}{d\mathbf{r}_i} = -2\left(\left|\mathbf{r}_{jk}\right|^2 + \left|\mathbf{r}_{ij}\right|^2 - 2\left|\mathbf{r}_{ki}\right|^2\right)\mathbf{r}_{ki} + 2\left|\mathbf{r}_{ki}\right|^2\mathbf{r}_{ij}. \tag{14}$$

$$\frac{d\lambda_3}{d\mathbf{r}_i} = 2\left(\left|\mathbf{r}_{jk}\right|^2 + \left|\mathbf{r}_{ki}\right|^2 - 2\left|\mathbf{r}_{ij}\right|^2\right)\mathbf{r}_{ij} - 2\left|\mathbf{r}_{ij}\right|^2\mathbf{r}_{ki}. \tag{15}$$

For $p = j$ we have,

$$\frac{d\lambda_1}{d\mathbf{r}_j} = 2\left(\left|\mathbf{r}_{ki}\right|^2 + \left|\mathbf{r}_{ij}\right|^2 - 2\left|\mathbf{r}_{jk}\right|^2\right)\mathbf{r}_{jk} - 2\left|\mathbf{r}_{jk}\right|^2\mathbf{r}_{ij}. \tag{16}$$

$$\frac{d\lambda_2}{d\mathbf{r}_j} = 2\left|\mathbf{r}_{ki}\right|^2\left(\mathbf{r}_{jk} - \mathbf{r}_{ij}\right). \tag{17}$$

$$\frac{d\lambda_3}{d\mathbf{r}_j} = -2\left(\left|\mathbf{r}_{jk}\right|^2 + \left|\mathbf{r}_{ki}\right|^2 - 2\left|\mathbf{r}_{ij}\right|^2\right)\mathbf{r}_{ij} + 2\left|\mathbf{r}_{ij}\right|^2\mathbf{r}_{jk}. \tag{18}$$

For $p = k$ we have,

$$\frac{d\lambda_1}{d\mathbf{r}_k} = -2\left(|\mathbf{r}_{ki}|^2 + |\mathbf{r}_{ij}|^2 - 2|\mathbf{r}_{jk}|^2\right)\mathbf{r}_{jk} + 2|\mathbf{r}_{jk}|^2\mathbf{r}_{ki}. \tag{19}$$

$$\frac{d\lambda_2}{d\mathbf{r}_k} = 2\left(|\mathbf{r}_{jk}|^2 + |\mathbf{r}_{ij}|^2 - 2|\mathbf{r}_{ki}|^2\right)\mathbf{r}_{ki} - 2|\mathbf{r}_{ki}|^2\mathbf{r}_{jk}. \tag{20}$$

$$\frac{d\lambda_3}{d\mathbf{r}_k} = 2|\mathbf{r}_{ij}|^2\left(-\mathbf{r}_{jk} + \mathbf{r}_{ki}\right). \tag{21}$$

Finally,

$$\frac{d\Lambda}{d\mathbf{r}_i} = -4\left(|\mathbf{r}_{jk}|^2 + |\mathbf{r}_{ij}|^2 - |\mathbf{r}_{ki}|^2\right)\mathbf{r}_{ki} + 4\left(|\mathbf{r}_{jk}|^2 + |\mathbf{r}_{ki}|^2 - |\mathbf{r}_{ij}|^2\right)\mathbf{r}_{ij}. \tag{22}$$

$$\frac{d\Lambda}{d\mathbf{r}_j} = 4\left(|\mathbf{r}_{ki}|^2 + |\mathbf{r}_{ij}|^2 - |\mathbf{r}_{jk}|^2\right)\mathbf{r}_{jk} - 4\left(|\mathbf{r}_{jk}|^2 + |\mathbf{r}_{ki}|^2 - |\mathbf{r}_{ij}|^2\right)\mathbf{r}_{ij}. \tag{23}$$

$$\frac{d\Lambda}{d\mathbf{r}_k} = -4\left(|\mathbf{r}_{ki}|^2 + |\mathbf{r}_{ij}|^2 - |\mathbf{r}_{jk}|^2\right)\mathbf{r}_{jk} + 4\left(|\mathbf{r}_{jk}|^2 + |\mathbf{r}_{ij}|^2 - |\mathbf{r}_{ki}|^2\right)\mathbf{r}_{ki}. \tag{24}$$

These expressions allow us to compute all derivatives in Eq. (7) and, in turn, the Jacobian in Eq. (4).

We still need to compute the derivative of the cell's area with respect to the positions of the vertices of the Voronoi cell. A straightforward calculation starting from Eq. (3) in the main text gives

$$\begin{aligned}
\left[\frac{\partial A_i}{\partial \mathbf{r}_\nu}\right]_k &= \left[\frac{\partial}{\partial \mathbf{r}_\nu}\left\{\frac{1}{2}\sum_{\mu\in\Omega_i}\left(\mathbf{r}_\mu \times \mathbf{r}_{\mu+1}\right)\cdot \mathbf{N}_i\right\}\right]_k \\
&= \left[\frac{1}{2}\frac{\partial}{\partial \mathbf{r}_\nu}\sum_{\mu\in\Omega_i}\varepsilon_{\alpha\beta\gamma}x_\beta^{(\mu)}x_\gamma^{(\mu+1)}N_\alpha^{(i)}\right]_k \\
&= \frac{1}{2}\sum_{\mu\in\Omega_i}\varepsilon_{\alpha\beta\gamma}N_\alpha^{(i)}\frac{\partial}{\partial x_k^{(\nu)}}\left(x_\beta^{(\mu)}x_\gamma^{(\mu+1)}\right) \\
&= \frac{1}{2}\varepsilon_{\alpha k\gamma}N_\alpha^{(i)}x_\gamma^{(\nu+1)} + \frac{1}{2}\varepsilon_{\alpha\beta k}N_\alpha^{(i)}x_\beta^{(\nu-1)} \\
&= \frac{1}{2}\varepsilon_{k\gamma\alpha}N_\alpha^{(i)}x_\gamma^{(\nu+1)} - \frac{1}{2}\varepsilon_{k\beta\alpha}N_\alpha^{(i)}x_\beta^{(\nu-1)} \\
&= \frac{1}{2}\left[\mathbf{r}_{\nu+1}\times\mathbf{N}_i - \mathbf{r}_{\nu-1}\times\mathbf{N}_i\right]_k,
\end{aligned} \tag{25}$$

where $\varepsilon_{\alpha\beta\gamma}$ is the Levi-Chivita symbol. Therefore, the expression for the area change due to displacing vertex i is

$$\left[\nabla_{\mathbf{r}_i}A_i\right]_k = \frac{1}{2}\sum_{\nu\in\Omega_i}\left[\mathbf{r}_{\nu+1}\times\mathbf{N}_i - \mathbf{r}_{\nu-1}\times\mathbf{N}_i\right]_m\left[\frac{\partial \mathbf{r}_\nu}{\partial \mathbf{r}_i}\right]_{mk}, \tag{26}$$

where, as above, we have assumed summation over the repeated index m. We finally derive the force on vertex i due to the area contractions,

$$\mathbf{F}_i^{area} = -\frac{1}{2}K_i\left(A_i - A_i^0\right)\sum_{\nu\in\Omega_i}\left[\mathbf{r}_{\nu+1}\times\mathbf{N}_i - \mathbf{r}_{\nu-1}\times\mathbf{N}_i\right]^T\left[\frac{\partial \mathbf{r}_\nu}{\partial \mathbf{r}_i}\right], \tag{27}$$

where T in superscript stands for transpose, i.e., $[\dots]^T[\dots]$ stands for a matrix product between a 1×3 matrix (i.e., a vector) and 3×3 Jacobian matrix.

We can now proceed to calculate derivatives of the second term in Eq. (1) in the main text. The perimeter of cell i is defined as

$$P_i = \sum_{\mu \in \Omega_i} |\mathbf{r}_{\mu+1} - \mathbf{r}_\mu| \,. \tag{28}$$

As above, we calculate

$$\begin{aligned}
\left[\frac{\partial P_i}{\partial \mathbf{r}_\nu}\right]_k &= \left[\frac{\partial}{\partial \mathbf{r}_\nu} \sum_{\mu \in \Omega_i} |\mathbf{r}_{\mu+1} - \mathbf{r}_\mu|\right]_k \\
&= \sum_{\mu \in \Omega_i} \frac{\partial}{\partial x_k^{(\nu)}} \left[\sum_\alpha \left(x_\alpha^{(\mu+1)} - x_\alpha^{(\mu)}\right)^2\right]^{1/2} \\
&= \sum_{\mu \in \Omega_i} \frac{1}{|\mathbf{r}_{\mu+1} - \mathbf{r}_\mu|} \sum_\alpha \left(x_\alpha^{(\mu+1)} - x_\alpha^{(\mu)}\right) \left(\delta_{\alpha k}\delta_{\nu,\mu+1} - \delta_{\alpha k}\delta_{\nu,\mu}\right) \\
&= \frac{x_k^{(\nu)} - x_k^{(\nu-1)}}{|\mathbf{r}_\nu - \mathbf{r}_{\nu-1}|} - \frac{x_k^{(\nu+1)} - x_k^{(\nu)}}{|\mathbf{r}_{\nu+1} - \mathbf{r}_\nu|}.
\end{aligned} \tag{29}$$

We therefore have

$$[\nabla_{\mathbf{r}_i} P_i]_k = \sum_{\nu \in \Omega_i} \left[\frac{x_m^{(\nu)} - x_m^{(\nu-1)}}{|\mathbf{r}_\nu - \mathbf{r}_{\nu-1}|} - \frac{x_m^{(\nu+1)} - x_m^{(\nu)}}{|\mathbf{r}_{\nu+1} - \mathbf{r}_\nu|}\right]_m \left[\frac{\partial \mathbf{r}_\nu}{\partial \mathbf{r}_i}\right]_{mk}, \tag{30}$$

where we also assume summation over the repeated index m. The force term resulting from perimeter contractions is then given as

$$\mathbf{F}_i^{perim} = -\Gamma_i P_i \sum_{\nu \in l_i} \left[\hat{\mathbf{r}}_{\nu,\nu-1} - \hat{\mathbf{r}}_{\nu+1,\nu}\right]^T \left[\frac{\partial \mathbf{r}_\nu}{\partial \mathbf{r}_i}\right], \tag{31}$$

where we have defined $\hat{\mathbf{r}}_{\nu,\nu-1} = \frac{\mathbf{r}_\nu - \mathbf{r}_{\nu-1}}{|\mathbf{r}_\nu - \mathbf{r}_{\nu-1}|}$, etc.

A similar calculation for the the last term in Eq. (1) in the main text leads to

$$\nabla_{\mathbf{r}_i} l_{\nu,\nu+1} = \sum_{\nu \in l_i} \left[\frac{\mathbf{r}_\nu - \mathbf{r}_{\nu-1}}{|\mathbf{r}_\nu - \mathbf{r}_{\nu-1}|} - \frac{\mathbf{r}_{\nu+1} - \mathbf{r}_\nu}{|\mathbf{r}_{\nu+1} - \mathbf{r}_\nu|}\right]^T \left[\frac{\partial \mathbf{r}_\nu}{\partial \mathbf{r}_i}\right],$$

where we have explicitly labelled the two nearest neighbours (in the counterclockwise direction as) as ν and $\nu + 1$. The force due to cell junction contractions is then computed as

$$\mathbf{F}_i^{junct} = -\sum_{\nu \in l_i} \left[\Lambda_{\nu-1,\nu} \frac{\mathbf{r}_\nu - \mathbf{r}_{\nu-1}}{|\mathbf{r}_\nu - \mathbf{r}_{\nu-1}|} - \Lambda_{\nu,\nu+1} \frac{\mathbf{r}_{\nu+1} - \mathbf{r}_\nu}{|\mathbf{r}_{\nu+1} - \mathbf{r}_\nu|}\right]^T \left[\frac{\partial \mathbf{r}_\nu}{\partial \mathbf{r}_i}\right]. \tag{32}$$

We now move to the second part, which is to determine the force on particle i as a result of displacing one of its surrounding particles. In order to do this, we go back to the original expression for the energy in the VM (Eq. (1) in the main text). The force on vertex i is then

$$\begin{aligned}
\mathbf{F}_i &= -\nabla_{\mathbf{r}_i} E_{VM} \\
&= -\nabla_{\mathbf{r}_i} \left\{\sum_{k=1}^N \left[\frac{K_k}{2}\left(A_k - A_k^0\right)^2 + \frac{\Gamma_k}{2} P_k^2\right] + \sum_{\langle \mu,\nu \rangle} \Lambda_{\mu\nu} l_{\mu\nu}\right\},
\end{aligned}$$

where N is the total number of cells. We first focus on the area and perimeter terms. We have

$$\begin{aligned}\mathbf{F}_i^{a+p} &= -\nabla_{\mathbf{r}_i}\sum_{k=1}^{N}\left[\frac{K_k}{2}\left(A_k - A_k^0\right)^2 + \frac{\Gamma_k}{2}P_k^2\right] \\ &= -\sum_{k=1}^{N_{part}}\sum_{\nu\in\Omega_k}\left(\nabla_{\mathbf{r}_\nu}\left[\frac{K_k}{2}\left(A_k - A_k^0\right)^2 + \frac{\Gamma_k}{2}P_k^2\right]\right)^T\left[\frac{\partial \mathbf{r}_\nu}{\partial \mathbf{r}_i}\right].\end{aligned}$$

Further, we have

$$\begin{aligned}\mathbf{F}_i^{a+p} &= -\sum_{k=1}^{N}\sum_{\nu\in\Omega_k}\left[K_k\left(A_k - A_k^0\right)\left(\nabla_{\mathbf{r}_\nu}A_k\right) + \Gamma_k P_k\left(\nabla_{\mathbf{r}_\nu}P_k\right)\right]^T\left[\frac{\partial \mathbf{r}_\nu}{\partial \mathbf{r}_i}\right] \\ &= -\sum_{k=1}^{N}\frac{K_k}{2}\left(A_k - A_k^0\right)\sum_{\nu\in\Omega_k}\left[\left(\mathbf{r}_{\nu+1}-\mathbf{r}_{\nu-1}\right)\times\mathbf{N}_k\right]^T\left[\frac{\partial \mathbf{r}_\nu}{\partial \mathbf{r}_i}\right] \\ &\quad -\sum_{k=1}^{N}\Gamma_k P_k\sum_{\nu\in\Omega_k}\left(\frac{\mathbf{r}_\nu-\mathbf{r}_{\nu-1}}{\left|\mathbf{r}_\nu-\mathbf{r}_{\nu-1}\right|}-\frac{\mathbf{r}_{\nu+1}-\mathbf{r}_\nu}{\left|\mathbf{r}_{\nu+1}-\mathbf{r}_\nu\right|}\right)^T\left[\frac{\partial \mathbf{r}_\nu}{\partial \mathbf{r}_i}\right].\end{aligned} \tag{33}$$

From the last expression it is clear that only vertices that are displaced by moving cell i are going to contribute to the force. These vertices are all "corners" of cell i and a subset of "corners" of its immediate neighbours affected by i. This gives us the algorithm for computing the total force on particle i coming from the area:

1. Loop over particle i and all its neighbours.
   (a) For particle i compute $\frac{K_i}{2}\left(A_i - A_i^0\right)$ and multiply it by the sum $\sum_{\nu\in\Omega_k}\left[\left(\mathbf{r}_{\nu+1}-\mathbf{r}_{\nu-1}\right)\times\mathbf{N}_i\right]^T\left[\frac{\partial \mathbf{r}_\nu}{\partial \mathbf{r}_i}\right]$. Note that this sum is over all vertices (corners) ν of the cell i.
   (b) For all immediate neighbours j of cell i compute $\frac{K_j}{2}\left(A_j - A_j^0\right)$ and multiply it with the sum $\sum_{\nu\in l_i\cap l_j}\left[\left(\mathbf{r}_{\nu+1}-\mathbf{r}_{\nu-1}\right)\times\mathbf{N}_j\right]^T\left[\frac{\partial \mathbf{r}_\nu}{\partial \mathbf{r}_i}\right]$. Note that $\nu\in l_i\cap l_j$ ensures that vertices ν surrounding j are taken into account only if they are affected by (and also belong to) cell i.

A similar algorithm can be used to compute force contribution of the perimeter term.

We now focus on the last term, which is the force along the cell junctions. If we note that we can write the cell junction term as

$$E^j = \sum_\mu \Lambda_{\mu,\mu+1}l_{\mu,\mu+1},$$

we have

$$\mathbf{F}_i^j = -\sum_{k=1}^{N}\sum_{\nu\in\Omega_k}\left[\Lambda_{\nu-1,\nu}\frac{\mathbf{r}_\nu-\mathbf{r}_{\nu-1}}{\left|\mathbf{r}_\nu-\mathbf{r}_{\nu-1}\right|}-\Lambda_{\nu,\nu+1}\frac{\mathbf{r}_{\nu+1}-\mathbf{r}_\nu}{\left|\mathbf{r}_{\nu+1}-\mathbf{r}_\nu\right|}\right]^T\left[\frac{\partial \mathbf{r}_\nu}{\partial \mathbf{r}_i}\right]. \tag{34}$$

As before, we loop over all vertices that are affected by changing the position of cell i, which are all corners of the cell i and a subset of corners of its immediate neighbours whose positions are determined by $\mathbf{r}_i$.

Combining Eqs. (33) and (34) leads to Eq. (7) in the main text. We note that in Eq. (34) there is no prefactor 2 (see last term in Eq. (1) in the main text) as the sum is over vertices of an individual cell.

Finally, we briefly address another contribution to the force which needs to be added to the model allow for simulations deep in the liquid-like phase. As discussed in Sec. "Activity driven fluidisation phase diagram" in the main text, similar to the SPV model, the AVM shows a transition between solid-like and fluid-like phases. In the solid-like phase, intercalation events are inhibited and without cell division and death, cells do not exchange their neighbours. In the fluid-like phase, on the other hand, cells are much more mobile and one observes a large number of T1 transitions and intercalations. In this regime, the Delaunay triangulation is very irregular with many obtuse triangles. If a triangle becomes very obtuse, its circumcenter is far

outside the triangle and even small changes in the position of one of its vertices leads to large movements of the circumcenter. For any simulation time step that is not extremely small, this can lead to unphysical self intersections of the triangulation and cause the simulation to be unreliable, or, in the most extreme cases, causes it to crash. In practice, in order to prevent this from happening while still being able to use a reasonably large time step, we endow each cell centre with a soft repulsive core of radius a. The repulsive potential between neighbouring cell centres is then given as

$$V_{soft}\left(r_{ij}\right) = \begin{cases} \frac{1}{2}k\left(r_{ij}-2a\right)^2 & \text{for } r_{ij} < 2a \\ 0 & \text{otherwise} \end{cases}, \tag{35}$$

where $r_{ij} = |\mathbf{r}_i - \mathbf{r}_j|$. This core prevents two cell centres from getting too close to each other. The corresponding force on cell i is

$$\mathbf{F}_i^{soft} = -\nabla_{\mathbf{r}_i} V_{soft}\left(r_{ij}\right) = \begin{cases} -k\left(r_{ij}-2a\right)\hat{\mathbf{r}}_{ij} & \text{for } r_{ij} < 2a \\ 0 & \text{otherwise} \end{cases} \tag{36}$$

with $\hat{\mathbf{r}}_{ij} \equiv |\mathbf{r}_i - \mathbf{r}_j| / r_{ij}$ and according to the Newton's third law, $\mathbf{F}_j^{soft} = -\mathbf{F}_i^{soft}$. The soft repulsion does not interfere with the AVM dynamics except for its regularising effect. In Fig. 6e-g in the main text, the pale spheres drawn at the cell centres have radius a, and they remain far removed from the cell boundaries.

We note that this repulsive force is similar in spirit to the limits that have to be imposed on edge lengths in order to prevent unphysical self-intersections in the triangulated models for lipid membranes, that have been extensively studied in the 1990s[1]. It is interesting to note that in the case of triangulated models for lipid membranes one also needs to introduce a maximum allowed edge length in order to prevent unphysical configurations. In the case of AVM, this is not necessary.

---

[1] Gompper G, Kroll D. Chapter 12. In: Nelson D, Piran T, Weinberg S, editors. Statistical Mechanics of Membranes and Surfaces. World Scientific Publishing; 2004. p. 359.
