# Supplementary material for: Active Vertex Model for cell-resolution description of epithelial tissue mechanics
Source: PLoS Comput Biol. 2017 Jun 30;13(6):e1005569. doi: 10.1371/journal.pcbi.1005569 (PMC5493290; doi:10.1371/journal.pcbi.1005569)
Supplement: S2 Appendix — (PDF) [file pcbi.1005569.s002.pdf]

Active Vertex Model for Cell-Resolution Description of Epithelial Tissue Mechanics

Daniel L. Barton, Silke Henkes, Cornelis J. Weijer and Rastko Sknepnek

S2 APPENDIX: ALGORITHM FOR HANDLING BOUNDARIES

Due to the dynamic nature of the model, even without cell division and death, it is not possible to retain a constant number of boundary particles. Instead, the boundary line has to be able to contract or extend in order to accommodate changes inside the tissue. This is achieved by dynamically adding and removing boundary particles.

We first focus on the boundary expansion. We require that all cells are contained within the boundary, that is, no dual vertices belonging to a cell are allowed to "spill" over the boundary line. This condition is violated if the angle opposite to a boundary edge is greater than 90°. In this case, the centre of the circumscribed circle falls outside the triangle and, therefore, outside the boundary. In order to prevent this from happening we perform the following check (see also Fig. 1):

1. For each boundary edge e compute angle α_e at the particle p_e opposite to it.

2. If $\alpha > 90°$

   (a) Compute the position, $\mathbf{r}_{p_n}$, of the new particle p_n by mirroring the coordinates of p_e, $\mathbf{r}_{p_e}$, with respect to edge e. If $\hat{\mathbf{r}}_e$ is the unit-length vector along edge e then

   $$\mathbf{r}_{p_n} = 2\left(\mathbf{r}_{p_e} \cdot \hat{\mathbf{r}}_e\right)\hat{\mathbf{r}}_e - \mathbf{r}_{p_e}. \quad (1)$$

   (b) Add a new boundary particle p_n at position $\mathbf{r}_{p_n}$ and mark it as boundary.

   (c) Remove boundary edge e, i.e., the two boundary particles at its end are no longer neighbours.

   (d) Connect p_n to the two boundary particles disconnected in (c).

   (e) Connect p_n to p_e.

3. If at least one new boundary particle was added in 2., rebuild the triangulation.

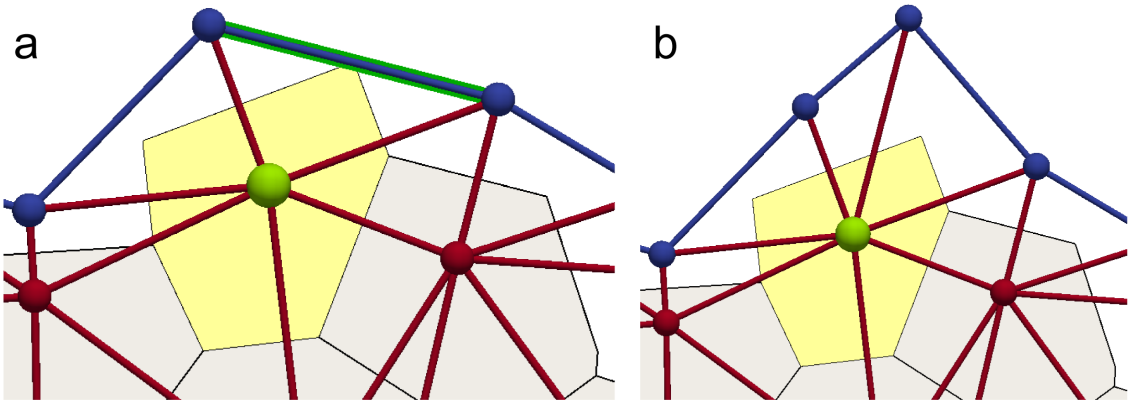

Figure 1. Expansion of the boundary by adding a new boundary particle. (a) If the angle at the internal particle shaded in green opposite to the highlighted edge reaches 90°, one of the corners of the cell (yellow polygon) touches the highlighted edge. This triggers a "flip" mechanism. (b) The internal particle shaded in green is mirrored along the shaded edge in (a) and a new boundary particle (top blue) is introduced. The shaded edge is flipped such that the new particle is connected to the "green" one.

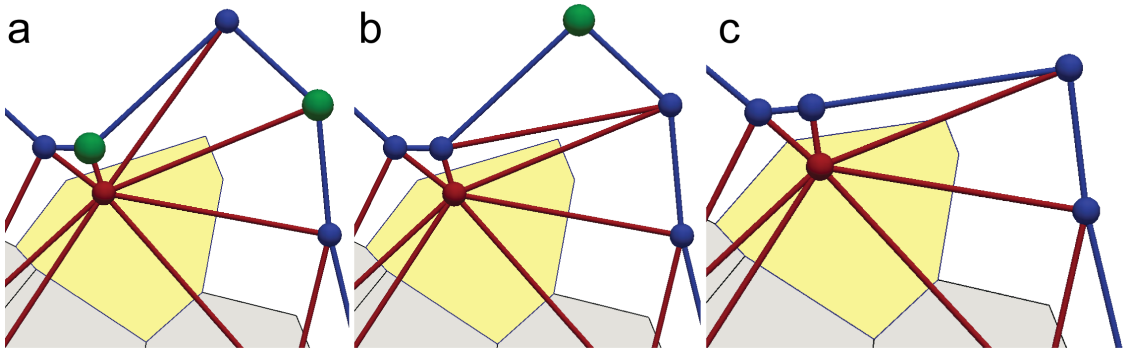

Figure 2. Shrinking of the boundary is achieved by removing boundary vertices that have only two bonds. (a) If the sum of angles at two particles shaded in green opposite the edge connecting the internal and boundary particles is greater than 180° the edge is flipped (this is the standard equiangulation move that occurs for all internal edges). (b) After the flip, the boundary particles shaded in green has only two bonds, both to its boundary neighbours. (c) The "green" particle in (b) is then removed.

Note that the procedure outlined above always converges in a single step.

Shrinking of the boundary is achieved by removing particles that have no connections to the internal particles. In this situation, no part of a cell can be inside a triangle that has two of its sides being boundary edges and it can be safely removed. The algorithm schematically outlined in Fig. 2 is as follows:

1. For each boundary particle p compute number of edges $n_e(p)$ that the particle belongs to.

2. If $n_e(p) \leq 2$ remove p.

Note that the position of the "dangling" particles does not directly affect the shape of the cell.

An important point to make here is that the algorithms used to add and remove boundary particles *do not* lead to sudden discontinuous changes of the shape of the cell or the force acting on its centre. However, addition and removal of boundary particles inevitably leads to discontinuous changes in the forces acting on boundary particles. A potential way to avoid such discontinuous behaviour would be to, e.g., smoothly "turn on" the interactions with newly added particles or by slowly "fade out" interaction with particles that are to be removed. In practice, however, the discontinuous causes by simply adding or removing boundary particles lead to changes in the force that are small and do not appreciably affect the simulation.
