# Supplementary material for: Active Vertex Model for cell-resolution description of epithelial tissue mechanics
Source: PLoS Comput Biol. 2017 Jun 30;13(6):e1005569. doi: 10.1371/journal.pcbi.1005569 (PMC5493290; doi:10.1371/journal.pcbi.1005569)
Supplement: S3 Appendix — (PDF) [file pcbi.1005569.s003.pdf]

Active Vertex Model for Cell-Resolution Description of Epithelial Tissue Mechanics

Daniel L. Barton, Silke Henkes, Cornelis J. Weijer and Rastko Sknepnek

S3 APPENDIX: IMPLEMENTATION

The AVM is implemented into the *SAMoS* code developed by this team[1]. In this appendix we first provide a general overview of the organisation of the *SAMoS* code and then discuss how the AVM in implemented in it.

SAMoS overview

SAMoS is a software package developed for simulating agent-based active matter systems confined to move on curved or flat surfaces[2]. The code is written in C++, using the C++98 standard with extensive use of the Standard Template Library and boost libraries[3]. It utilises a modular, object oriented design making it very flexible and simple to extend. It adheres to modern software design principles and uses a cross-platform build system (*cmake*) as well as automatic documentation generation with *Doxygen*[4].
SAMoS consists of several components each implemented as class hierarchies.

- **System** - central component that handles the system configuration;
- **Parser** - a recursive descent parser for parsing files that control execution of the simulation (configuration files). It is implemented using boost's Phoenix library[5];
- **Messenger** - logs system messages (warnings, errors, etc.) as well as meta-data (parameter trees in JSON or XML format) for data curation;
- **Neighbour list** - handles build and update of the Verlet neighbour list[6];
- **Constraint** - handles projection on various flat or curved surfaces. It is possible to have multiple constraints acting on different groups of particles;
- **Interactions** - handles all interactions on and between particles. Interaction parameters can be type-specific, i.e., it is possible to simulate multicomponent systems. It is also possible to have multiple interaction types simultaneously present in the system;
  - **Pair/multi-body** interactions - handles all interactions that involve pairs or multiplets of particles (Vertex, Lennard-Jones, soft repulsion, Morse, etc.);
  - **External** interactions - handles all forces that act on a single particle, such as external fields and/or activity;
  - **Bond/angle** interactions - handles interaction between connected beads for simulating filaments;
- **Alignment** - handles alignment of the particle orientation
  - **Pair** alignment - handles various models for alignment to the direction of neighbouring particles;
  - **External** alignment - handles alignment to internal or external cues, such as velocity, cell shape or external fields;
- **Dump** - handles output of the system snapshot in various formats (VTP, raw text data file, input configuration for restarts, etc.). It is possible to have multiple dump types present in the same simulation;
- **Log** - handles output of the current system state, such as total potential energy, mean velocity, temperature, etc.;

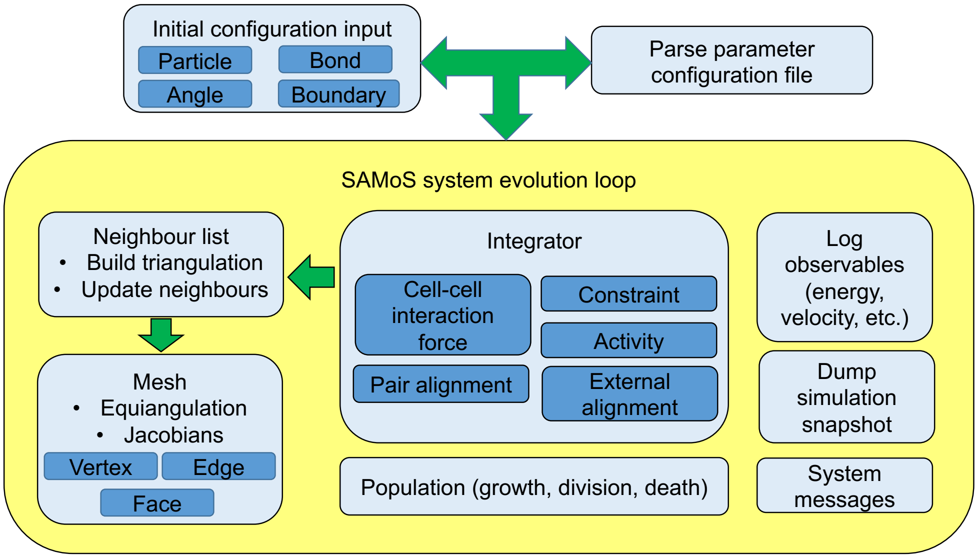

Figure 1. Overview of the general organisation of the AVM implementation into *SAMoS*.

- **Population** - handles addition and removal of particles, such as during cell division and death. It is possible to have multiple population controls acting at the same time or on different groups of particles;

- **Integrator** - handles various numerical integrators (Langevin, Brownian, NVE, etc.) for solving the equations of motion. Different integrators can acts on different groups of particles (e.g. no motion, for keeping a subset of particles fixed).

The components are designed to be as loosely coupled as possible, in order to ensure flexibility, ease of testing and debugging; and also to make extensions of the code, such as adding a new interaction force or population control mechanism, as simple as implementing a new subclass with a minimal need to modify the core of the code.

In order to perform a simulation with *SAMoS*, the user has to supply two or three text files: 1) a file, referred to as the *data* file, containing the initial configuration of the system (i.e the initial positions and velocities of the particles, particle types, polarity, etc.), 2) for AVM simulations only, a file, referred to as the *boundary* file, containing the initial labels and connectivity of the boundary particles and 3) the parameter file, referred to as the *configuration* file, which sets the simulation protocol (interaction types and parameters, constraints, type and frequency of dumps, simulation time step, etc.). Commands in the configuration file are parsed and executed in the order they appear. Examples of data and configuration files can be found in the *configurations* directory in the *SAMoS* installation.

In the current implementation, *SAMoS* runs on a single CPU core.

AVM implementation

The AVM is implemented as an extension of the *SAMoS* code. The main addition to the code involves a light-weight implementation of the half-edge data structure[7] as a separate, *Mesh* class. This class holds the information about the Delaunay triangulation and computes its dual Voronoi diagram. The *Mesh* class also ensures that the Delaunay character of the triangulation is preserved between rebuilds using the equiangulation procedure discussed in Sec. “Maintaining a Delaunay triangulation/Voronoi tessellation” in the main text. Finally, *Mesh* computes the Jacobian matrix, $\left[\frac{\partial \mathbf{r}_\nu}{\partial \mathbf{r}_i}\right]$ and supplies its elements to the part of

the code that computes the force on the cells. A feedback loop from the integrator ensures that the *Mesh* class always has the correct position of the cell centres.

The Delaunay triangulation of the initial positions of the cell centres is performed using CGAL's Delaunay library[8]. In order to properly include the boundary of the cell sheet, we compute a constrained Delaunay triangulation[9], where the boundary line, supplied by the user as an input file, acts as the constraint placed on the triangulation. It is important to note that all Delaunay triangulation libraries always produce a convex hull of the region that needs to be triangulated. The existence of the boundary line and the use of the constrained triangulation method allows us to simply and clearly distinguish between the triangles that are "inside" the tissue and should be kept and the "outside" ones that are an artefact of the triangulation procedure and should be discarded. This weeding out of outside triangles imposes some performance burden on the computation and we do not perform it at every time step, but maintain it to be Delaunay by applying equiangulation moves that are fast to compute. The triangulation is completely rebuilt only after the steps that involve cell division/death events or boundary extension/contraction. In order not to disturb the connectivity of the boundary, only the internal edges can be flipped. In practice, depending on the parameter values, the triangulation is rebuilt once every 10-50 time steps. This substantially improves the performance of the code.

The force on the cell centre, Eq. (7) in the main text, is computed by a subclass of the pair/multi-body submodule in the Interactions module discussed in the previous section. This class invokes the *Mesh* class in order to obtain the information about positions of dual vertices and components of the Jacobian matrix.

An overview of the general organisation of the AVM implementation is shown in Fig. 1.

[1] Soft Active Matter on Surfaces (SAMoS);. Available from: `https//github.com/sknepneklab/SAMoS`.

[2] Sknepnek R, Henkes S. Active swarms on a sphere. Physical Review E. 2015;91(2):022306.

[3] `http://www.boost.org/`;.

[4] van Heesch D. Doxygen;. Available from: `http://www.stack.nl/~dimitri/doxygen/index.html`.

[5] de Guzman J, Marsden D, Heller T, Fletcher J. Boost Phoenix Parser;. `http://www.boost.org/doc/libs/1_61_0/libs/phoenix/doc/html/index.html`.

[6] Allen MP, Tildesley DJ. Computer simulation of liquids. Oxford university press; 1989.

[7] Campagna S, Kobbelt L, Seidel HP. Directed edges - A scalable representation for triangle meshes. Journal of Graphics tools. 1998;3(4):1–11.

[8] Project TC. CGAL User and Reference Manual. 4th ed. CGAL Editorial Board; 2016. Available from: `http://doc.cgal.org/4.9/Manual/packages.html`.

[9] Chew LP. Constrained delaunay triangulations. Algorithmica. 1989;4(1-4):97–108.
